# Fluorescence cryo-microscopy: current challenges and prospects

**DOI:** 10.1016/j.cbpa.2014.05.007

**Published:** 2014-06

**Authors:** Rainer Kaufmann, Christoph Hagen, Kay Grünewald

**Affiliations:** 1 Division of Structural Biology, Wellcome Trust Centre for Human Genetics, University of Oxford, Roosevelt Drive, Oxford OX3 7BN, UK; 2 Department of Biochemistry, University of Oxford, South Parks Road, Oxford OX1 3QU, UK

## Abstract

Studying biological structures with fine details does not only require a microscope with high resolution, but also a sample preparation process that preserves the structures in a near-native state. Live-cell imaging is restricted mostly to the field of light microscopy. For studies requiring much higher resolution, fast freezing techniques (vitrification) are successfully used to immobilize the sample in a near-native state for imaging with electron and X-ray cryo-microscopy. Fluorescence cryo-microscopy combines imaging of vitrified samples with the advantages of fluorescence labeling of biological structures. Technical considerations as well as the behavior of fluorophores at low temperatures have to be taken into account for developing or adapting super-resolution methods under cryo conditions to exploit the full potential of this technique.

## Highlights

• CryoFM allows imaging of vitrified biological samples with fluorescence microscopy.
• There are significant challenges to achieve high-resolution cryoFM imaging.
• Fluorophore characteristics at low temperature offer additional advantages.
• Cryo super-resolution fluorescence imaging will give dramatic resolution improvement.

**Current Opinion in Chemical Biology** 2014, **20**:86–91

This review comes from a themed issue on **Molecular Imaging**

Edited by **Christian Eggeling** and **Mike Heilemann**

For a complete overview see the Issue and the Editorial

Available online 19th June 2014

**http://dx.doi.org/10.1016/j.cbpa.2014.05.007**

1367-5931/© 2014 The Authors. Published by Elsevier Ltd. This is an open access article under the CC BY license (http://creativecommons.org/licenses/by/3.0/)

## Introduction

Fluorescence cryo-microscopy (cryoFM) originates from various fields of research and is motivated by a range of biological, chemical and physical questions. First ‘cryo’-microscopy was performed when imaging snowflakes in the 19th century (for review see [1]). Almost half a century ago liquid nitrogen cooled and temperature regulated sample stages for light microscopes have been developed to study thawing processes along applications in the biomedical field [2,3]. In contrast, the motivation of performing measurements at low temperature in the field of single molecule spectroscopy is very different. Spectral lines of single molecules become extremely narrow at low temperatures, revealing much more detailed information about themselves and their interaction with external stimuli [4], but also allowing detection of very weak single molecule effects [5^•^]. Correlative cryo-microscopy is a relatively recent development of imaging the same sample with different imaging modalities such as fluorescence, X-ray and/or electron cryo-microscopy. This allows combining visualization of ultrastructural details with the molecular specificity of fluorescence labeling [6–10]. Moving to low temperatures in this field of cryoFM is primarily motivated by the fact that the sample needs to be kept in amorphous ice to maintain structural preservation in a near-native state across all imaging modalities. The decreased photo-bleaching at lower temperatures [4] is merely a welcome side effect.

**Figure 1:**
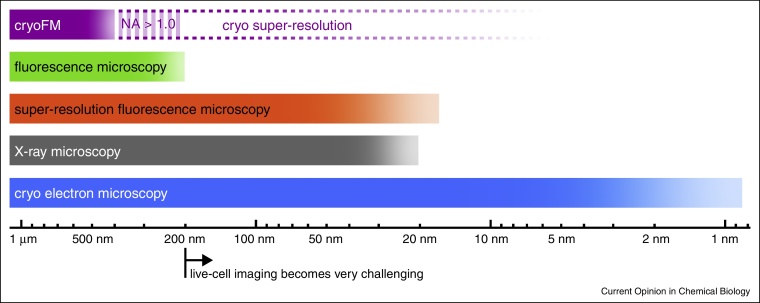
Resolution scale. Colored bars represent the resolution achieved with the according microscopy techniques in biological samples. Opaque color corresponds to a range routinely accessible, best values that have been achieved but are not routine are indicated by increased transparency. Super-resolution fluorescence microscopy already reaches a similar range as X-ray microscopy for chemically fixed samples, but remains very challenging for live-cell imaging. CryoFM, which also provides the possibility of imaging biological structures in a near-native state, is currently limited in resolution even more than conventional fluorescence microscopy. The development of cryo immersion objectives and the adaptation of super-resolution methods for cryo conditions will lead to a dramatic increase of the resolution in cryoFM (indicated by dashed lines).

CryoFM is becoming more and more popular in the field of correlative cryo-microscopy. Here, the demand of improved resolution far below the diffraction limit of light is evident when comparing with its counterparts in electron and X-ray cryo-microscopy (Figure 1). Likewise is the ability to image cryo immobilized biological samples in a near-native state with fluorescence microscopy an emerging driving force toward super-resolution cryoFM. We will discuss advantages and challenges of cryoFM based on the current state of this technique with a distinct focus on the prospects of super-resolution fluorescence microscopy under cryo conditions.

## Advantages and current challenges in cryoFM

Cryo-microscopy in general allows imaging biological structures in a near-native state. At ambient temperatures only living cells provide unperturbed structural details. Fluorescence microscopy techniques provide live-cell imaging capabilities, but the resolution is restricted to ∼200 nm. Only the application of super-resolution methods [11^•^] allows overcoming the diffraction limit, but this remains very challenging for imaging living cells [12–14]. For achieving a substantially improved resolution, in most cases movement of structures needs to be stopped. This typically requires chemical fixation of the sample which can cause structural changes in the sample [15]. In contrast, cryo-immobilization using rapid freezing techniques (vitrification) preserves the structures in a near-native state in glass-like amorphous ice. This procedure is frequently applied for imaging fine structural details with electron or X-ray cryo-microscopy [16–18]. In fluorescence microscopy the benefits of vitrified samples are currently not fully exploited due to the very limited resolution of optical setups for cryoFM. In the first instance, this results from the lack of appropriate immersion objectives dedicated for cryo conditions which restricts the numerical aperture (NA) of the imaging system and thereby the resolution to a range of 400–500 nm. Additionally, super-resolution methods, which have been developed for fluorescence microscopy at ambient temperatures, have so far not been adapted to cryo conditions. Changed photophysics of fluorescent molecules at low temperatures might be both, beneficial as well as challenging for super-resolution cryoFM.

## Fluorophore behavior at low temperatures

A big issue in fluorescence microscopy at ambient temperatures is photo-bleaching which often hampers specific experiments. The two major mechanisms leading to irreversible bleaching of fluorescent molecules are suppressed at cryo temperatures [4]. Transformational changes, which are often crucial steps on the way to photodecomposition of the fluorescent molecule, are reduced [19]. The diffusion of small reactive molecules such as oxygen is arrested and thus bleaching via photo-oxidation of fluorescent molecules is suppressed as well [4]. It has been shown that the number of photons emitted by fluorescent molecules at low temperatures can be increased up to two orders of magnitude compared to ambient temperatures [20]. This effect has also been shown for fluorescent proteins in vitrified cells in comparison to living cells [6,7,9].

On the other hand, the signal to noise ratio of fluorescence imaging at low temperatures can be dramatically reduced due to high triplet population of the fluorescent molecules [21,22]. A study with organic dyes reported a triplet population of 80–90% at 76 K, corresponding to a reduction of brightness of almost 10 times [22]. In this case triplet depopulation was possible by additional illumination of the molecules with an appropriate wavelength to reestablish nearly the original signal to noise ratio [22].

Photo-switching or blinking of fluorescent proteins and organic dye molecules, an effect well studied at ambient temperatures [23^••^,24,25], is still present at low temperatures [26–29,30^•^]. Weisenburger *et al.* recently showed reversible photo-switching of single organic dye molecules at 4.4 K with bright and dark states lasting many seconds up to minutes [30^•^,31]. Long-lived dark states in organic fluorophores are reached via the triplet state [28]. Their life-time shows almost no temperature dependency, but the lack of oxygen can substantially decelerate the recovery to the fluorescent ground state [28]. Fluorescent proteins can be switched with moderate to high excitation intensities to a reversibly bleached state from which they recover to the fluorescent state spontaneously or photoinduced [26,29]. Photo-switching at low temperatures is here facilitated by photoinduced protonation rather than conformational changes (e.g. isomerization) which play a competing role at ambient temperatures [29]. Future studies will have to address this at the single molecule level to gain a more detailed understanding of the different pathways of reversible and irreversible photo-bleaching at low temperatures. Switching efficiency, life-times of bright and dark states as well as background characteristics (population of molecules which are not switching/blinking) are crucial points for the development or adaptation of super-resolution methods in cryoFM (for more detailed discussion see section ‘*Prospects for super-resolution cryoFM*’).

Probably the most important characteristic of fluorescent molecules at low temperatures for the field of single molecule spectroscopy are the very narrow absorption and emission spectra [4]. But also in fluorescence microscopy the temperature dependency of the molecules spectra could be utilized for distinguishing different fluorophore types in cryoFM whose spectra would overlap at ambient temperatures (e.g. improved multi-color measurements).

## Different implementations for cryoFM

**Figure 2:**
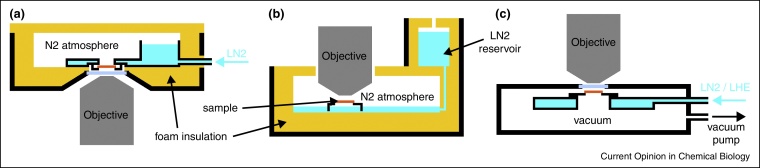
Schematic overview of different stand-alone cryo stage and cryostat designs for cryoFM. **(a)** Cryo stage design for an inverted microscope setup. Cooling is achieved by pumping liquid nitrogen (LN2) through the cryo stage. Imaging is performed through a long working distance air objective which is separated from the cryo environment by a glass window and kept at ambient temperature (for details see [45]). **(b)** Cryo stage for an upright microscope configuration [10] with an autonomous LN2 cooling mechanism. The objective is dipping into the cold nitrogen atmosphere inside the cryo stage. An air objective with a long working distance is required to avoid heat transfer to the sample. Additional cooling of the objective allows reducing the working distance and thus an objective with a larger NA can be used to increase resolution [36]. Full integration of the objective into the cryo stage and immersion imaging under cryo conditions have been shown with a design of overall similar principle [9]. **(c)** Vacuum insulated cryostat. Temperature stability and range (also liquid helium (LHE) cooling possible) is increased compared to cryo stages as shown in (a) and (b), but the implementation of a sample transfer mechanism is very complicated. Higher NA air objectives can be used if placed inside the cryostat, but the NA is limited to <1.0 due to the incompatibility of cryo immersion with vacuum [37,38^••^].

A crucial factor besides the photophysical considerations for cryoFM imaging is the design of the optical system. Most setups developed for cryoFM originated from the field of correlative cryo-microscopy [32^••^]. The main advantage of these implementations is the ability for transferring vitrified samples. Therein, the samples have to be kept below the devitrification temperature of ∼135 K to maintain the structural preservation [33], thus precooling of the cryo stage is required before insertion of the sample. Additionally, a transfer system allows extraction of the sample for subsequent electron or X-ray microscopy imaging. The typical design of cryo stages for correlative applications consist of an insulated liquid nitrogen cooled chamber, that can be opened for sample exchange, and a long working distance air objective that is kept at ambient temperature either via separation by a glass window or via a temperature gradient from the sample (Figure 2a,b). Thus the NA of the optical system is limited to <1.0, typically ∼0.8. This restricts the resolution to a range of 400–500 nm. Additionally the number of detectable photons is reduced by almost a factor of 2 compared to high NA oil immersions objectives used for fluorescence microscopy at ambient temperatures. One implementation has shown the principle feasibility of immersing an oil objective with an NA of 1.3 into a cryogen in a liquid nitrogen cooled optical setup [9]. However, neither the resolution nor the imaging quality achieved with this system has been reported quantitatively. The application of tomographic imaging to cryoFM allows 3D isotropic resolution [34^•^]. Another challenge for cryoFM is the stability of a dedicated cryo stage. Small reservoirs of liquid nitrogen, connections to supply hoses and temperature gradients between different components of the cryo stage make these designs very susceptible for having mechanical instabilities while imaging. This can become a problem for precise correlative measurements or in the case of more advanced cryoFM applications (e.g. co-localization studies) or super-resolution techniques.

Closed and vacuum insulated systems (Figure 2c), which are mainly used in single molecule spectroscopy, can offer much better temperature and mechanical stabilities [30^•^]. This is of particular importance when long exposure times are needed to detect very weak signals or to obtain very high precision for the position determination of ‘point-like’ objects in cryoFM such as virus particles or even single fluorescent molecules [30^•^,35,36]. Cryostats that offer a very high imaging stability usually do not have the possibility of a transfer system for imaging vitrified samples [37,38^••^]. The integrations of objectives and optical imaging paths in the column of a transmission electron microscope [8] or X-ray microscope [17], which were already equipped with sample transfer systems, represent approaches of a thermally stable fluorescence cryo-imaging system. They are beneficial for correlative cryo-microscopy from a sample handling point of view, but the NA of the optical imaging system is further reduced by spatial restrictions inside the column, limiting the resolution even more than compared to setups for cryoFM with objectives outside the cryo chamber.

## Prospects for super-resolution cryoFM

Currently, the major drawback in cryoFM is the relatively low resolution. The development of a dedicated cryo immersion objective to reach an NA above 1.0 and thereby a resolution comparable to applications at ambient temperatures is one of the most important requirements. This will be dependent on how well an objective can be designed and built for operation under cryo conditions without creating strong aberrations due to different thermal expansion coefficients of the different elements in the objective.

In parallel, super-resolution methods might be adapted to cryo conditions to overcome the diffraction limit in cryoFM. Here, the mechanical stability of the system will be of greatest importance as the image acquisition takes substantially longer than for basic fluorescence imaging. Recently, the feasibility of reaching a stability with a sample drift in the range of 100 nm per hour has been reported [30^•^].

The foundation of most super-resolution methods, which have been developed for fluorescence microscopy at ambient temperatures, is the photo-switching of fluorophores [39] used for labeling the structures or proteins of interest. As discussed above, various studies have been performed to investigate photo-switching of fluorescent proteins and organic dye molecules at low temperatures.

Methods based on single molecule localization [40] are dependent on the time the fluorescent molecules remain in the bright and the dark state. It has been shown that single molecule localization accuracy in the subnanometer range can be achieved using photo-switching of isolated organic dye molecules with relatively long life-times of the bright state in conjunction with suppressed photo-bleaching in cryo conditions [30^•^]. However, only if the life-time of the dark state is much longer than the life-time of the bright state, densely located single molecule signals can be separated from each other for a precise position determination, necessary for super-resolution imaging. The development of dedicated fluorophores exploiting the specific photophysics under cryo condition, optimizing the fluorophore micro-environment or using imaging conditions that would provide a slowed-down switching cycle (longer life-times of bright and dark state) might permit a higher photon yield per single molecule blinking event while still keeping them separable from each other. This could lead to the development of cryo single molecule localization microscopy with a resolution exceeding those of super-resolution techniques currently applied in fluorescence microscopy at ambient temperatures.

The resolution of stimulated emission depletion (STED) microscopy is dependent on the efficiency of the stimulated emission process [41]. At ambient temperatures the anti-stokes excitation, which arises from the occupancy of high excited vibrational states of the molecules ground state, competes with the stimulated emission, thereby reducing the STED efficiency and eventually restricting the achievable resolution [42]. The temperature dependency of the occupation of high excited vibrational states allows a reduction of the anti-stokes excitation at low temperatures. A first proof of principle experiment of STED microscopy at 76 K of fluorescent microspheres has already shown a resolution increase by a factor of 1.6 compared to ambient temperatures [22].

Structured illumination microscopy (SIM) [43,44] is a super-resolution microscopy method which is not based on photo-switching or photophysical transitions of the fluorescent molecules. The major limitation of this technique is photo-bleaching during data acquisition, especially for live-cell imaging which is its biggest strength as the resolution improvement is limited by diffraction to a factor of two. SIM could greatly benefit from the suppressed photo-bleaching of fluorophores at cryo-conditions when biological structures could be studied in a near-native state in vitrified cells. For this application again a cryo immersion objective with high NA is critical to reach a resolution better than conventional fluorescence microscopes do at ambient temperatures for live-cell imaging.

## Conclusion

Especially the field of correlative cryo-microscopy would greatly benefit in case the resolutions of the different imaging techniques would match each other more closely. However, cryoFM also offers the possibility to study immobilized biological samples in a near-native state. As the resolution in FM is currently not yet in the range at which structural changes associated with chemical fixation are visible, so far only electron and X-ray microscopy are broadly exploiting imaging vitrified biological samples. The development of cryo immersion objectives, but especially the development or adaptation of super-resolution techniques for cryo conditions will increase the resolution in cryoFM dramatically. CryoFM might currently be at a turning point from being a niche application mainly motivated by basic correlative purposes to becoming a much more powerful technique on its own.

## References and recommended reading

Papers of particular interest, published within the period of review, have been highlighted as:

• of special interest
•• of outstanding interest

## Note added in proof

Recently, two papers provided new insight into the prospects of single molecule localization microscopy under cryo conditions. Chang *et al.* [46] showed that certain photo-activatable fluorescent proteins maintain their switching possibility at low temperature allowing determination of single molecule positions. Kaufmann *et al.* [47] demonstrated super-resolution imaging of structures labeled with standard fluorescent proteins in vitrified cells improving the resolution of fluorescence cryo-microscopy by a factor of 3-5.
